# Impacts of the apoptosis inhibitor of macrophage (AIM) on obesity-associated inflammatory diseases

**DOI:** 10.1007/s00281-013-0405-5

**Published:** 2013-11-27

**Authors:** Satoko Arai, Toru Miyazaki

**Affiliations:** 1 Laboratory of Molecular Biomedicine for Pathogenesis, Center for Disease Biology and Integrative Medicine, Faculty of Medicine, The University of Tokyo, 7-3-1 Hongo, Bunkyo-ku, Tokyo, 113-0033 Japan; 2 CREST, Japan Science and Technology Agency, 7-3-1 Hongo, Bunkyo-ku, Tokyo, 113-0033 Japan

**Keywords:** AIM (apoptosis inhibitor of macrophage), Chronic inflammation, Lipolysis, IgM, Autoantibody

## Abstract

Obesity is associated with various metabolic and cardiovascular diseases caused by chronic, low-grade inflammation that is initially observed in obese adipose tissue. In addition, many etiological studies in humans have shown a strong correlation between obesity and inflammatory autoimmune diseases. In this review, we focus on the involvement of apoptosis inhibitor of macrophage (AIM), a macrophage-derived blood protein, in both types of immune response. Through differential mechanisms, AIM thereby plays key roles in the pathogenesis of atherosclerosis, metabolic diseases, and obesity-associated autoimmune diseases. Thus, the regulation of blood AIM levels or AIM function has the potential to serve as a next-generation therapy against these inflammatory diseases brought about by modern lifestyle.

## Obesity-associated inflammatory diseases in modern society

The prevalence of obesity is rapidly increasing due to drastic changes in lifestyle, particularly eating habits. Obesity is closely associated with insulin resistance, which triggers and/or accelerates multiple metabolic disorders including type 2 diabetes, cardiovascular diseases, and fatty liver dysfunction. It is widely known that insulin resistance is caused by chronic, low-grade inflammation in obese adipose tissue [1–5]. This subclinical state of inflammation is dependent mainly on the innate immune system through the activation of Toll-like receptors (TLR) expressed on adipocytes by fatty acids, a process which leads to the production of inflammatory adipokines and the recruitment of classically activated inflammatory macrophages (M1 macrophages) into obese adipose tissue [6–8]. Lean adipose tissue contains a resident population of alternatively activated macrophages (M2 macrophages), which can suppress the inflammatory response induced by both adipocytes and macrophages partly via the secretion of interleukin-10. Hence, obesity induces a switch in the macrophage activation state in adipose tissue towards M1-polarization, which subsequently leads to inflammation [9–12].

In addition to metabolic and cardiovascular diseases, many etiological and clinical studies in humans have shown a strong correlation between obesity and autoimmune diseases. These conditions are largely accompanied by increased levels of autoantibodies such as diabetes-associated antibodies against pancreatic β-cell antigens (e.g. insulin, glutamic acid decarboxylase (GAD), and protein tyrosine phosphatase-like protein, IA2), chronic thyroiditis-associated anti-thyroid peroxidase or anti-thyroglobulin antibody, and infertility-associated anti-sperm antibody [13–17]. In addition, pathogenic immunoglobulin (Ig) G antibodies, including a unique profile of autoantibodies, have been found in obese humans and mice [18].

The association between obesity and inflammatory diseases can be attributed to two distinct immunological responses: chronic inflammation through stimulating innate immunity leading to insulin resistance and activation of a humoral immune response that triggers autoantibody production. In this review, we discuss the pathogenesis of obesity-associated inflammatory diseases from the immunological perspective by focusing on the apoptosis inhibitor of macrophage (AIM, also known as Spα and CD5L) [19]. We initially identified AIM as an apoptosis inhibitor that supports the survival of macrophages against various apoptosis-inducing stimuli [19]. However, our recent studies revealed that AIM is involved in the progression of both types of obesity-associated inflammatory response though differential mechanisms.

## Apoptosis inhibitor of macrophage

AIM protein is a secreted protein of the scavenger receptor cysteine-rich superfamily [20]. Although the protein sequences of human and mouse AIM are well conserved, large differences exist in the glycosylation states; mouse AIM is heavily glycosylated with *N*-glycans, whereas human AIM is not *N*-glycosylated. We previously demonstrated that such a *N*-glycosylation state influences the activity and secretion efficiency of AIM protein [21].

AIM is produced solely by tissue macrophages under transcriptional regulation by nuclear receptor liver X receptor/retinoid X receptor (LXR/RXR) heterodimers [19, 22–24] and is therefore expressed in lipid-laden macrophages in atherosclerotic lesions. We demonstrated that AIM induction is associated with atherosclerogenesis by supporting the survival of macrophages within lesions [24]. Indeed, atherosclerotic plaques were markedly reduced in size in mice doubly deficient for AIM and the low-density lipoprotein (LDL) receptor (*AIM* ^*−/−*^ *LDL* ^*−/−*^) compared with *AIM* ^*+/+*^ *LDL* ^*−/−*^ mice fed a high-cholesterol diet [24, 25].

As a secreted molecule, AIM is detected at varying levels in human and mouse blood [26–32]. Interestingly, serum AIM increased with the progression of obesity in mice fed a high-fat diet (HFD) [31]. Other studies have suggested that AIM is multifunctional and effective in cell types other than macrophages, including B and natural killer T lymphocytes [33–35]. In addition, Lozano's group reported that AIM attaches to certain bacteria and induces their coagulation [36]. This “sticky” characteristic is a hallmark of scavenger receptor cysteine-rich superfamily proteins [20, 37–39].

## AIM induces lipolysis in adipocytes suppressing an increase in fat mass

In addition to its apoptosis inhibitory effect, we found that AIM induces lipolysis in adipose tissue. When differentiated 3T3-L1 adipocytes in culture were challenged with AIM, the size and the number of lipid droplets of triacylglycerol within the adipocytes markedly decreased [31]. Through this AIM-induced lipolytic response, a certain amount of glycerol and free fatty acids (FFA), the constituents of triacylglycerol, were effluxed from the cells [40, 41]. In support of these in vitro observations, production of both visceral and subcutaneous fat tissue was accelerated in *AIM* ^*−/−*^ mice fed a HFD (60 % fat) compared with *AIM* ^*+/+*^ mice fed the same diet. In addition, basal levels of serum FFA and glycerol were lower in obese *AIM* ^*−/−*^mice than in obese *AIM* ^*+/+*^ mice [31]. These differences in *AIM* ^*−/−*^ and *AIM* ^*+/+*^ mice were corrected by the intraperitoneal administration of recombinant AIM [31]. Interestingly, both obese *AIM* ^*−/−*^ mice and *AIM* ^*+/+*^ mice showed comparable metabolic parameters (e.g., body temperature, oxygen consumption, and food intake) and locomotor activity [31]. Thus, AIM influences adipose tissue mass, which essentially regulates fat and body weight, through specifically affecting adipocytes.

Interestingly, unlike most ligands in the blood, such as cytokines and growth factors, which bind to specific receptors and mediate signal transduction to affect their target cells, blood AIM is incorporated into adipocytes via endocytosis mediated by the CD36 scavenger receptor and functions directly in the cytosol of the target cells [31]. Such direct functioning in the absence of signaling is unusual in secreted molecules, with only a limited number of reported examples, including fibroblast growth factors-1 and -2 [42, 43] and epidermal growth factor [44], in which the cytosolic delivery of exogenous proteins was shown to mediate the biological effects in mammalian cells, and also in some plant and bacterial toxins [45, 46]. In addition, some exogenous antigens in dendritic cells can access the cytosol via machinery similar to that for intracellular transport where they are presented by major histocompatibility complex class I molecules [47, 48]. The mechanism responsible for AIM translocation from the endosomal compartment into the cytosol remains unknown.

## Two independent modes of lipolysis induction

Lipolysis usually occurs during periods of energy deprivation. Under fasting conditions, increased amounts of catecholamine are released from the hypothalamus and bind to the β-adrenergic receptor, thereby mediating the cyclic adenosine monophosphate (cAMP)-dependent signaling cascade. This response phosphorylates protein kinase A (PKA), which activates hormone-sensitive lipase (HSL) and increases the levels of the *adipose triglyceride lipase (ATGL)* mRNA [49–55].

In contrast, AIM does not mediate signals. Despite lipolytic consequences, no HSL phosphorylation was observed in AIM-treated adipocytes in vitro [31, 56]. In vivo phosphorylation of HSL or its upstream PKA in epididymal adipose tissue was not enhanced in obese wild-type mice compared with lean mice, although lipolysis was apparently enhanced, given the elevated serum levels of FFA and glycerol [56]. Similarly, *AIM* ^*−/−*^ mice fed a HFD showed no increase in HSL phosphorylation. In addition, forced induction of lipolysis in obese *AIM* ^*−/−*^ mice by intravenous injection of AIM activated neither HSL nor PKA phosphorylation in epididymal adipose tissue [56]. In accordance with these observations, increased HSL and PKA phosphorylation levels were comparably detected in the epididymal adipose tissue of *AIM* ^*−/−*^ and *AIM* ^*+/+*^ mice in response to 24-h fasting [56].

In the cytosol of adipocytes, incorporated AIM associates with fatty acid synthase (FASN), which is highly expressed in adipose tissue and catalyzes the synthesis of saturated fatty acids, such as palmitate, from acetyl-CoA and malonyl-CoA precursors [31, 56]. This binding of AIM remarkably reduces the enzymatic activity of FASN [31, 56]. Consistent with this finding, FASN activity was significantly increased in the epididymal fat of *AIM* ^*−/−*^ mice compared with *AIM* ^*+/+*^ mice and was subsequently decreased following direct injection of AIM. Since treatment of adipocytes with AIM or the FASN inhibitor C75 has similar lipolytic consequences, the lipolytic effect of AIM on adipocytes is likely due to the suppression of FASN activity [31, 56]. Thus, two distinct modes of lipolysis occur in different physiological situations: catecholamine-dependent lipolysis under fasting conditions and AIM-induced lipolysis under obese conditions.

## AIM targets lipid droplet-coating proteins via regulating PPARγ activity

Numerous studies have suggested that polyunsaturated fatty acids and related molecules can activate peroxisome proliferator-activated receptor (PPAR)γ, a master transcription factor for the differentiation of adipocytes, although the identity of the biological ligand(s) for PPARγ has not been elucidated [57–60]. Metabolomics analysis revealed that the proportion of palmitic acid (C16:0), the primary product synthesized by FASN, was significantly reduced in adipocytes treated with AIM. Similarly, the proportions of multiple saturated fatty acids harboring longer chains such as stearic acid (C18:0) and related unsaturated fatty acids are also reduced in response to AIM [56].

**Fig. 1:**
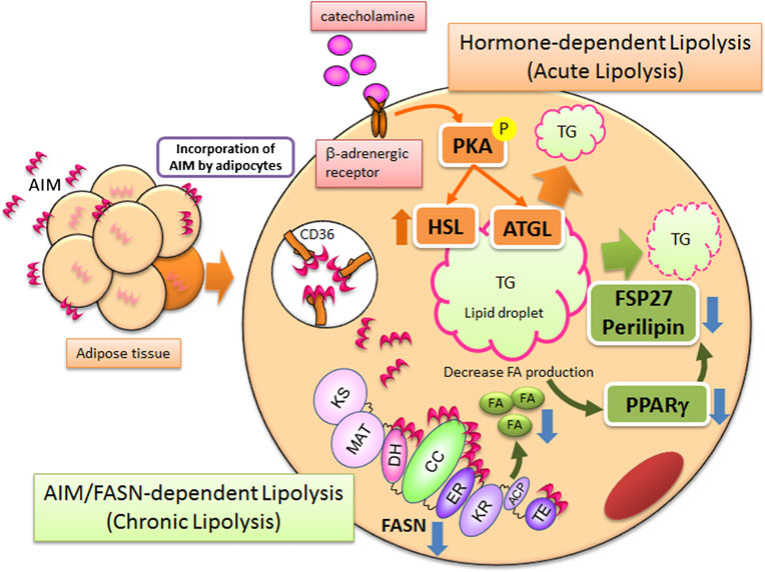
Two modes of lipolysis distinguished by AIM. Catecholamine mediates signals thereby activates and/or augments lipases which directly degrade TG, leading to lipolysis. In contrast, AIM decreases lipid droplet-coating proteins via reducing FASN activity, without influencing lipases

Interestingly, the transcription of different lipid coating genes, including *FSP27* and *Perilipin*, whose mRNA levels decrease in response to AIM in adipocytes [31], is directly regulated by PPARγ [61, 62]. It is plausible then that suppression of FASN activity by AIM reduces the production of PPARγ biological ligand(s), thereby decreasing the transcriptional activity of PPARγ and resulting in downregulation of the droplet-coating gene expression that leads to lipolysis (Fig. 1). We corroborated this idea as follows [56]. First, we assessed whether the presence of rosiglitazone, a selective PPARγ agonist, or T0070907, a selective PPAR antagonist, influenced the lipolytic effect of AIM in 3T3-L1 adipocytes. Several parameters with remarkable involvement in AIM-induced lipolysis (i.e. increased glycerol efflux, downregulation of *FSP27* and *Perilipin* mRNA levels, and increased inflammatory gene expression) were inhibited by the presence of rosiglitazone in a dose-dependent fashion. In contrast, a synergistic effect of these lipolytic consequences was detected following combined administration of recombinant AIM and T0070907. Second, we assessed the effect of AIM on the transcriptional activity of PPARγ more directly by creating 3T3-L1 adipocytes stably transfected with a luciferase reporter gene conjugated with a PPAR-binding element (PPRE) at the 5' end [57]. Challenge with AIM significantly decreased the luciferase activity in a dose-dependent fashion, as with T0070907. In addition, the luciferase activity induced by rosiglitazone was significantly suppressed by AIM.

## AIM-induced lipolysis mediates migration of macrophages into adipose tissue

The suppressed lipolytic state of adipose tissue in *AIM* ^*−/−*^ mice [31] results in more advanced adipocyte hypertrophy than in *AIM* ^*+/+*^ mice, and the overall mass of visceral fat and body weight is markedly greater [31]. It is interesting to note, however, that the obesity-associated infiltration of inflammatory macrophages (M1 macrophages) into adipose tissue was dramatically suppressed in *AIM* ^*−/−*^ mice compared with *AIM* ^*+/+*^ mice after being fed a 12-week HFD [63]. In addition, the administration of AIM to obese *AIM* ^*−/−*^ mice resulted in an accumulation of M1 macrophages in adipose tissue [63]. Thus, the presence of AIM is indispensable for obesity-associated recruitment of adipose tissue macrophages. However, AIM exhibits no chemoattractive activity in a macrophage migration assay. By contrast, conditioned medium from adipocytes that had been challenged with AIM efficiently attracted macrophage cells [63]. Furthermore, conditioned medium from adipocytes treated with AIM in the presence of a CD36-neutralizing antibody to inhibit AIM-dependent lipolysis [31] did not efficiently attract macrophages, suggesting that AIM-induced lipolysis in adipocytes is responsible for macrophage recruitment.

Previous studies have demonstrated that saturated fatty acids activate TLR4 and that this response is tightly associated with obesity-induced inflammation [64–68]. Thus, it is plausible that an increase in blood AIM induces vigorous lipolysis in obese adipose tissue and that saturated fatty acids effluxed from adipocytes as a result of lipolysis might activate chemokine production in adipocytes via the stimulation of TLR4 in a paracrine/autocrine fashion [69–71]. Indeed, palmitic acid and stearic acid, the major fatty acids comprising triglyceride droplets [72] and well-known stimulators of TLR4 [18, 68, 73, 74], were identified as the components released by adipocytes in response to AIM-induced lipolysis. Consistent with this finding, conditioned medium from adipocytes treated with AIM efficiently activated the TLR signaling cascade in adipocytes, inducing the degradation of I-kappa-B-alpha (IκBα) and the production of chemokines such as monocyte chemotactic protein (MCP)-1, chemokine (C-C motif) ligand 5/RANTES, MCP-2, and MCP-3. Similar effects of TLR activation and chemokine production were observed when 3T3-L1 adipocytes were treated with palmitic acid and stearic acid [63]. Similarly, when AIM was injected into wild-type or *TLR4* ^*−/−*^ mice, induction of chemokine mRNA was significantly less efficient compared with wild-type mice, although lipolysis was induced in both wild-type and *TLR4* ^*−/−*^ mice, as shown by the increased serum FFA and glycerol levels [63].

**Fig. 2:**
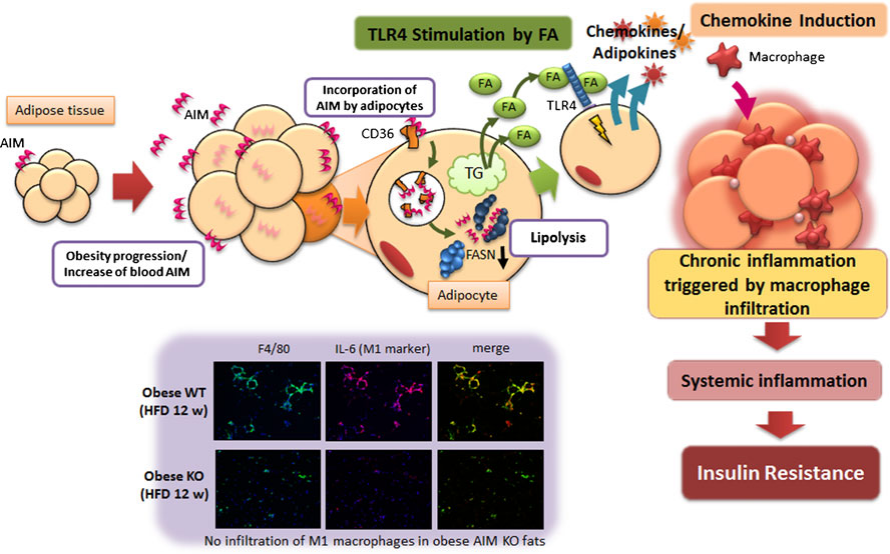
A scheme of how excess of AIM-dependent lipolysis induces adipose tissue macrophage recruitment. No inflammatory macrophage infiltration in fat tissue in obese *AIM* ^*−/−*^ mice (*lower photos*)

Taken together, lipolysis induced by increased blood AIM under obese conditions releases large amounts of saturated fatty acids from adipocytes. This response stimulates chemokine production in adipocytes via TLR4 activation, resulting in M1 macrophage migration (Fig. 2).

## Absence of inflammation and insulin resistance in obese *AIM*^*−/−*^ mice

The progression of obesity-associated inflammation is prevented both locally and systemically in obese *AIM* ^*−/−*^ mice due to the abolished infiltration of inflammatory macrophages. Accordingly, substantial insulin-stimulated phosphorylation of AKT/ protein kinases protein kinase B and glycogen synthase kinase 3-beta [75] was observed in adipose tissue, skeletal muscle (the gastrocnemius), and liver of *AIM* ^*−/−*^ mice in contrast to the markedly diminished phosphorylation in *AIM* ^*+/+*^ mice [63]. Thus, insulin sensitivity was maintained in obese *AIM* ^*−/−*^ mice. Similarly, whole-body glucose intolerance and insulin resistance observed in obese *AIM* ^*+/+*^ mice were ameliorated in obese *AIM* ^*−/−*^ mice, as shown by intraperitoneal glucose and insulin tolerance tests [63]. Thus, the absence of AIM apparently prevents insulin resistance under obese conditions.

## AIM and autoimmune-susceptible natural IgM

Another recent topic regarding AIM is its involvement in obesity-associated autoimmune diseases. As briefly described earlier, it is well known that obesity in humans often increases the serum levels of multiple autoantibodies, definitively causing autoimmune diseases. However, the elements involved in this autoimmune process and the overall contribution of obesity to autoantibody production remain unclear.

Due to the germline V gene segment, a large proportion of natural IgM is polyreactive to not only foreign antigens but also autoantigens, including nucleic acids, heat shock proteins, carbohydrates, and phospholipids [76–78]. Thus, IgM is believed to be important for the progression of autoimmunity. Moreover, natural IgM has a relatively low antigen-binding affinity that is compensated for by the pentameric nature of secreted IgM. This forms an immune complex (IC) with antigens and the complement component C3 which is subsequently deposited on splenic follicular dendritic cells (FDC) [79–82]. Antigen presentation by FDC ICs to follicular B cells is required for the development of long-lived plasma cells that produce high-affinity IgG [83]. Therefore, IgM-IC bound to autoantigens should stimulate the autoantibody response that mediates the progression of autoimmunity.

Interestingly, the potential association between AIM and natural IgM in human blood has been suggested, although its physiological significance is entirely unknown [84]. Indeed, when size-fractionated wild-type mouse serum was assessed for the presence of AIM and IgM, the fractions containing AIM and IgM overlapped precisely at a high molecular weight (>500 kDa), suggesting that most circulating AIM is associated with IgM pentamers [32]. Experiments with different monoclonal IgM clones revealed that AIM likely binds to the Fc region, since AIM binds to IgM regardless of the type of variable region. In contrast, AIM does not bind to IgG in vivo or in vitro.

## Association between AIM and IgM maintains blood AIM levels

Before discussing autoimmunity, we would like to briefly mention the beneficial association between IgM and AIM. A strong correlation between AIM and natural IgM levels in the blood has been found in both humans and mice. Accordingly, the serum AIM level was far lower in mice lacking blood IgM, such as secreted-type IgM-deficient (Δsμ) mice [85], than in wild-type mice, although the AIM mRNA level in macrophages was comparable in various tissues in all types of mice [32]. Hence, the association between AIM and IgM increases the protein stability of AIM in the blood. In agreement with these findings, intravenous injection of monoclonal mouse IgM rapidly increased serum AIM levels in Δsμ mice.

The mechanism of how the association between IgM and AIM stabilizes blood AIM levels is as follows: Free AIM is excreted in the urine, but this response is prevented when AIM forms a complex with IgM, resulting in accumulation of AIM in the blood [32]. This complex formation maintains the blood AIM level at a relatively high concentration (~10 μg/ml). Indeed, when AIM was injected into mice doubly deficient for sμ and AIM (Δsμ *AIM* ^*−/−*^) and into *AIM* ^*−/−*^ mice, the decrease in serum AIM levels was more prominent in the Δsμ *AIM* ^*−/−*^ mice. In parallel, AIM excretion in the urine was notably higher in Δsμ *AIM* ^*−/−*^. By contrast, AIM did not appear to contribute to the protein stability of IgM as both *AIM* ^*+/+*^ and *AIM* ^*−/−*^ mice showed comparable levels of blood IgM.

## AIM involvement in IgM-dependent antibody maturation in the spleen

It is well known that IgM-IC is deposited on splenic FDC through an interaction between the complement component C3 within the complex and the FDC complement receptor (CD21/CD35), and presents antigens to germinal center (GC) B cells [79–83]. Interestingly, IgM-IC remains on the FDC surface for a long time, which increases the probability of coming into contact with highly matched B-cell receptor-bearing GC-B cells. Such contact induces differentiation into mature plasma cells.

When spleen specimens from wild-type mice were stained for AIM, IgM, and FDC, accumulations of both AIM and IgM were specifically observed in FDC within splenic GCs [32]. Notably, this co-existence of AIM is required for the retention of IgM-IC with antigens on the FDC surface [32] and has been corroborated by two sets of experiments. First, IgM alone or in association with recombinant AIM (IgM/AIM) was intravenously injected into Δsμ *AIM* ^*−/−*^ mice, and the presence of IgM on the FDC cell surface was tested kinetically. Injection of IgM alone showed no significant deposition on the FDC surface, whereas injected of IgM/AIM revealed profoundly increased FDC IgM levels. The increase was still obvious 48 h after the injection. Second, the retention of antigens on the FDC surface was tested using the 2,4,6-trinitrophenyl (TNP) antigen, which was shown to be bound to IgM. When TNP conjugated with Ficoll was injected into *AIM* ^*+/+*^ and *AIM* ^*−/−*^ mice, the TNP antigen was maintained on the FDC surface more efficiently in *AIM* ^*+/+*^ mice than in *AIM* ^*−/−*^ mice. Consistent with this finding, the splenic FDC area stained positive for TNP 48 h after the injection in both mice strains, but there was markedly less staining in *AIM* ^*−/−*^ mice.

## AIM interferes with the binding of IgM to the Fcα/μ receptor

Then, what is the mechanism of how AIM supports the retention of IgM-IC on the FDC surface? We focused on the Fcα/μR, the Fc-receptor for both IgM and IgA [86], because Fcα/μR expression is detected mainly on the FDCs [86, 87] and the Fcα/μR induces internalization of IgM, thereby reducing IgM retention on the cell surface [86]. To analyze the influence of AIM on IgM binding to the Fcα/μR, we treated HEK293T cells expressing the Fcα/μR with a monoclonal IgM with or without AIM association. Flow cytometry showed that the association between AIM and IgM markedly decreased the binding of IgM to the Fcα/μR. Similarly, Fcα/μR-expressing HEK293T cells incubated with serum from *AIM* ^*+/+*^ mice showed reduced surface staining for IgM compared with cells incubated with *AIM* ^*−/−*^ serum. Consistent with the binding results, incorporation of IgM by the cells through the Fcα/μR was also drastically disturbed by the association of AIM. Thus, AIM interferes with the binding of IgM to the Fcα/μR and its internalization through antagonizing the receptor. Taken together, it is likely that the presentation of IgM-dependent antigens on the surface of splenic FDC to GC-B cells is deficient in *AIM* ^*−/−*^ mice due to rapid internalization of IgM-IC via the Fcα/μR.

## B-cell TLR4 mediates the obesity-associated increase in natural IgM levels

As expected from the correlation between AIM and natural IgM levels in the blood, IgM levels were markedly increased in line with AIM levels [31] in wild-type mice fed a HFD. Evidence suggests that stimulation of cell surface TLR4 activates splenic marginal zone B cells, a major producer of natural IgM [88–90], and subsequently induces high amounts of polyclonal IgM production in an antigen-independent fashion [91, 92]. Indeed, no significant increase in blood IgM levels was observed in *TLR4* ^*−/−*^ mice fed a HFD for 6 weeks, suggesting that the increase in natural IgM levels in obese mice was brought about by the stimulation of TLR4 expressed on B cells. It is very likely that increased levels of fatty acids, which are effluxed from obese adipocytes and/or directly supplied by a HFD, may activate B-cell TLR4 [64].

## Obesity-associated autoantibody production is supported by AIM

**Fig. 3:**
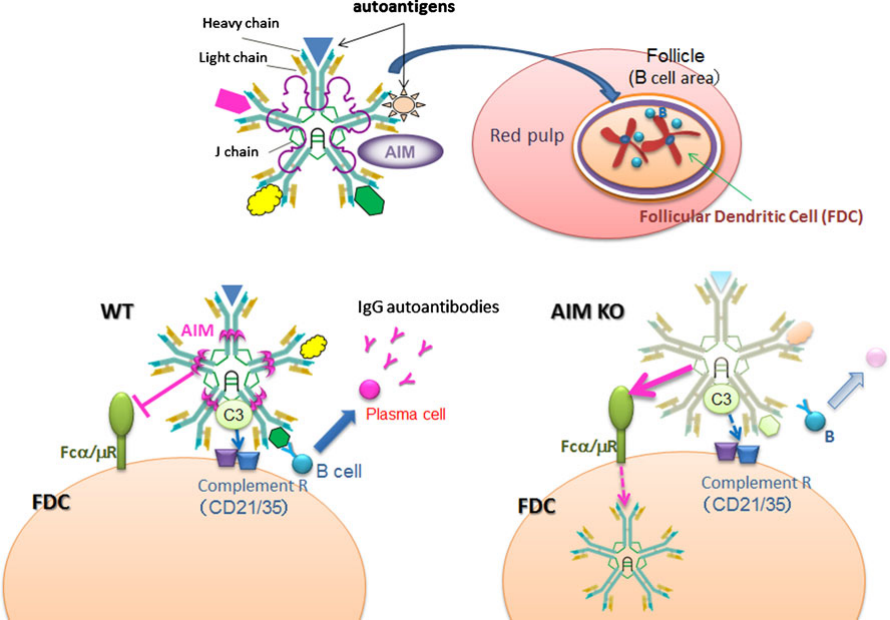
The role of AIM in obesity-associated autoantibody production. In blood, AIM is associated with IgM pentamer, and thus translocates to splenic GC with IgM/autoantigen complex (*upper scheme*). There, AIM supports the retention of IgM on the FDC cell surface by interfering with the IgM incorporation into FDC which is mediated by Fcα/μ receptor. This results in efficient autoantigen presentation to GC-B cells, leading to development of high-affinity autoantibody producing plasma cells (*WT*). In contrast, in the absence of AIM, the IgM/autoantigen complex is internalized by Fcα/μ, resulting in less efficient autoantigen presentation, overall leading to suppression of autoimmunity (*KO*)

Because of the self-reactive nature of natural IgM, its augmentation might stimulate IgG autoantibody production in obese mice. This was tested in a proteome microarray containing 70 autoantigens [93–95], using the serum from obese wild-type mice. Compared with lean mice, serum from mice fed a HFD for 12 weeks contained significantly increased levels of IgG autoantibodies against more than 30 variable autoantigens related to DNA, U1RNP, histone, SSA/SSB, and the cell matrix. In contrast, serum from *AIM* ^*−/−*^ mice fed a HFD for the same period revealed markedly lower levels of IgG antibodies against most of the autoantigens to which the *AIM* ^*+/+*^ serum responded [32]. Consistent with these findings, flow cytometry showed a decreased number of long-lived plasma cells [96], which produce high-affinity IgGs, in the bone marrow of obese *AIM* ^*−/−*^ mice compared with obese *AIM* ^*+/+*^ mice. Thus, the lack of AIM annuls the IgM-dependent maturation of high-affinity IgG-producing plasma B cells, tempering obesity-associated IgG autoantibody production (Fig. 3).

## Conclusion

In this article, we have reviewed the key roles of AIM in controlling the progression of multiple obesity-associated inflammatory diseases. The relationship between obesity and blood AIM levels is analogous to that of the accelerator and brake in a car. The more speed (body fat) we gain, the more braking (blood AIM) we need to reduce the speed to keep the car under control. In this regard, AIM is beneficial for impeding the progression of obesity. Under “severely obese” conditions, however, body fat and blood AIM behave as if the accelerator and brake are applied simultaneously at very high intensity: such a situation will cause extensive damage to the car. Similarly, severe damage will occur to our body. Excess fatty acids are effluxed from adipocytes due to excessive AIM-induced lipolysis. This process stimulates adipocyte TLR4 levels, which results in the release of chemokines, which in turn recruits inflammatory macrophages into the adipose tissue, leading to insulin resistance. In this regard, AIM is detrimental for metabolic disorders. Thus, during the early phases of metabolic syndrome prior to developing prominent obesity with limited lipid storage in adipocytes, AIM can prevent the progression of obesity via lipolysis, while the reduction of blood AIM levels or inhibition of AIM function will protect very obese individuals from developing metabolic inflammatory diseases such as diabetes, cardiovascular diseases, and autoimmune diseases.

**Fig. 4:**
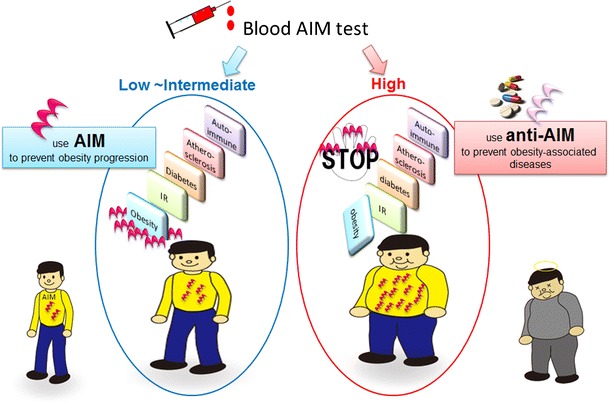
To prevent obesity-associated inflammatory diseases

To conclude, the combined application of AIM (i.e., AIM agonists) and anti-AIM (i.e., AIM antagonists) has the potential to serve as a next-generation therapy for preventing harmful obesity-associated inflammatory diseases brought about by modern lifestyles (Fig. 4). We are currently conducting large-scale cohort studies to determine the blood AIM levels in both healthy individuals and patients with various diseases. We anticipate that our results will be useful for establishing the threshold levels of blood AIM, which will subsequently help us decide whether an AIM agonist or AIM antagonist should be used.
